# Dynamic Resistance Training Improves Cardiac Autonomic Modulation and Oxidative Stress Parameters in Chronic Stroke Survivors: A Randomized Controlled Trial

**DOI:** 10.1155/2019/5382843

**Published:** 2019-11-20

**Authors:** Bruno Bavaresco Gambassi, Hélio José Coelho-Junior, Camila Paixão dos Santos, Ivan de Oliveira Gonçalves, Cristiano Teixeira Mostarda, Emanuele Marzetti, Samir Seguins Sotão, Marco Carlos Uchida, Kátia De Angelis, Bruno Rodrigues

**Affiliations:** ^1^School of Physical Education, University of Campinas (UNICAMP), Campinas, SP, Brazil; ^2^Ceuma University, São Luis, MA, Brazil; ^3^Department of Geriatrics, Neurosciences and Orthopedics, Teaching Hospital “Agostino Gemelli”, Catholic University of the Sacred Heart, Rome, Italy; ^4^Department of Physiology, Federal University of São Paulo (UNIFESP), São Paulo, SP, Brazil; ^5^Center of Health Sciences, University of Mogi das Cruzes (UMC), Mogi das Cruzes, SP, Brazil; ^6^Physical Education Department, Federal University of Maranhão (UFMA), São Luis, MA, Brazil

## Abstract

Stroke survivors are at substantial risk of recurrent cerebrovascular event or cardiovascular disease. Exercise training offers nonpharmacological treatment for these subjects; however, the execution of the traditional exercise protocols and adherence is constantly pointed out as obstacles. Based on these premises, the present study investigated the impact of an 8-week dynamic resistance training protocol with elastic bands on functional, hemodynamic, and cardiac autonomic modulation, oxidative stress markers, and plasma nitrite concentration in stroke survivors. Twenty-two patients with stroke were randomized into control group (CG, *n* = 11) or training group (TG, *n* = 11). Cardiac autonomic modulation, oxidative stress markers, plasma nitrite concentration, physical function and hemodynamic parameters were evaluated before and after 8 weeks. Results indicated that functional parameters (standing up from the sitting position (*P* = 0.011) and timed up and go (*P* = 0.042)) were significantly improved in TG. Although not statistically different, both systolic blood pressure (Δ = −10.41 mmHg) and diastolic blood pressure (Δ = −8.16 mmHg) were reduced in TG when compared to CG. Additionally, cardiac autonomic modulation (sympathovagal balance–LF/HF ratio) and superoxide dismutase were improved, while thiobarbituric acid reactive substances and carbonyl levels were reduced in TG when compared to the CG subjects. In conclusion, our findings support the hypothesis that dynamic resistance training with elastic bands may improve physical function, hemodynamic parameters, autonomic modulation, and oxidative stress markers in stroke survivors. These positive changes would be associated with a reduced risk of a recurrent stroke or cardiac event in these subjects.

## 1. Introduction

Stroke, a neurological disease commonly caused in response to abnormal blood perfusion of the brain tissue, is the leading cause of permanent disability worldwide [1]. Neuromuscular impairments, such as muscle loss, dynapenia, and reduced muscle power are commonly observed in patients with stroke and represent a crucial risk factor for the development of limited physical function, disability, and poor prognosis [2–4].

In addition to the neuromuscular alterations, marked oxidative stress, impairment in blood pressure control mechanisms (e.g., baroreflex sensitivity), and severe autonomic dysfunction, characterized by an elevated sympathetic activity, combined with a reduced or unchanged parasympathetic activity [5–7] might also been observed in stroke survivors and collaborated to genesis of cardiovascular complications (e.g., hypertension and myocardial infarction) in this population [8].

On the other hand, the practice of physical exercise has been considered an effective nonpharmacological strategy for poststroke individuals, since it mitigates physical, neurological, and cardiovascular sequelae. Indeed, prior studies have found improved cardiovascular health and physical function in stroke survivors after exercise training protocols [9, 10].

Nevertheless, researchers have argued that resistance training (RT), a type of physical exercise in which muscle contractions occur against a predetermined load [11], should receive priority attention in rehabilitation protocols for stroke survivors to maximize gains in mobility and independence [12]. A recent review of our group [12] indicated that the benefits of RT in stroke survivors go beyond the neuromuscular system and may include improvements in anxiety levels and quality of life.

However, most studies investigated RT protocols and stroke were based on exercise and isokinetic machines, limiting their external validity [12, 13]. Besides that, evidence for the effects of RT on cardiac autonomic modulation and oxidative stress markers in stroke survivors are still scarce.

Based on these premises, the present study investigated the impact of an 8-week dynamic RT protocol with elastic bands on the physical function, hemodynamic parameters, cardiac autonomic modulation, oxidative stress markers, and plasma nitrite concentration in stroke survivors. We hypothesized that all these parameters may be improved in response to our protocol of dynamic RT.

## 2. Materials and Methods

### 2.1. Experimental Design

This is an interventional, controlled, randomized study conducted upon approval by the São Judas Tadeu University Ethical Committee (São Paulo, SP, Brazil) (CAAE: 64859916.0.0000.0089). The study was conducted according to the Declaration of Helsinki and registered in the Brazilian database of clinical trials (Register ID: U1111-1202-8242; 26/09/2017).

### 2.2. Participants

Participants were recruited by convenience from the rehabilitation center of the Albert Sabin Municipal Physiotherapy Center located in Poá, Brazil. Prior to recruitment, volunteers of the present study were participating of a physical activity program, which aimed to restore social life and increase individual's levels of physical movement. The program was offered to those patients who have finished the neurological poststroke rehabilitation program but stayed at home for a long time and were not able to reestablish the same performance of activities of daily living (ADL) and social life that they had prior stroke. Motivational and religious dialogues were proposed at the beginning and end of each session. Physical movements aimed to stimulate body movement were performed with the individual's sitting in a chair for 25-30 minutes without external load. Movements included put arms and legs up, down, forward, and backward, rotate the trunk to the right and left sides, and move the trunk forward and backward. Individuals who did not want or could not perform the exercises were not discouraged from attending the sessions and were common to observe that some of them went to the sessions to talk to other people. Most individuals were from low-income families and were taken to the rehabilitation center by a minibus offered by the city hall. Sessions occurred twice a week for 40-50 minutes under the supervision of a physical educator. A washout period of 4 weeks separated was concluded prior to baseline evaluations.

Subjects were eligible to take part of the present study if they: (a) aged 45-75 years; (b) were able to walk with or without a walking aid; (c) were independent to perform basic activities of daily living, according to Barthel index [14]; (d) had a clinical diagnosis of stroke confirmed by computed tomography or magnetic resonance imaging at least 6 months prior to enrollment; (e) lived in the community; and (f) completed a standard neurological poststroke rehabilitation program. Candidate participants were excluded if they were not able to sign the informed consent form, had history of smoking or alcohol abuse in the last 6 months, had history of uncontrolled hypertension and/or diabetes mellitus according to medical records, used beta blockers, showed disabling pain during exercise, were incapable to perform exercise sessions and/or any of the evaluations (self-reported), and not attended at least 90% of training sessions. Participants had not been engaged in regular exercise training programs during the previous 6 months, according to the Baecke Habitual Physical Activity Questionnaire [15], and no changes in dose and drug classes were registered during the protocol.

**Figure 1:**
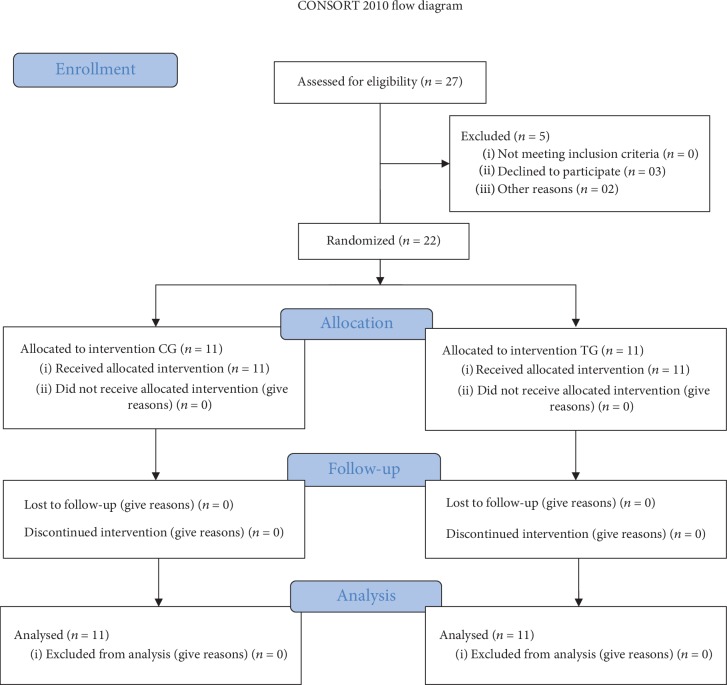
CONSORT flow diagram.

Twenty-seven stroke patients were enrolled in this study and five subjects were excluded. Twenty-two consenting patients were randomized 1 : 1 into the control group (CG, *n* = 11) and trained group (TG, *n* = 11) (Figure 1).

### 2.3. Resistance Training (RT) Intervention

**Figure 2:**
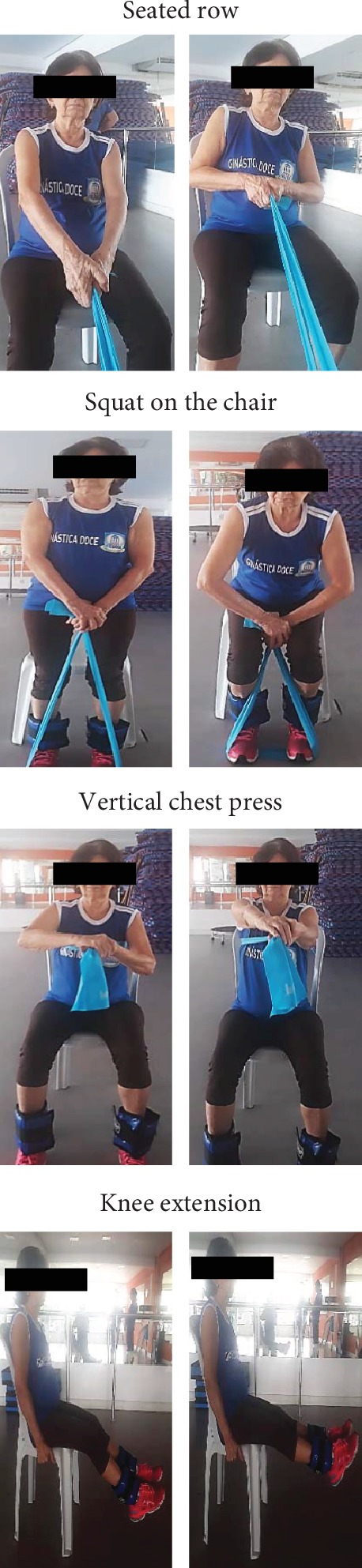
Representation of resistance training protocol execution.

The dynamic RT protocol was performed two times per week over an 8-week period with a 48 h rest interval provided between each exercise session. Resistance exercises were performed using elastic bands [16] (Thera Band®, Ohio, USA) and ankle wrist weights. The physical exercises were performed in the following order: (1^st^) seated row, (2^nd^) squat on the chair, (3^rd^) vertical chest press, and (4^th^) knee extension (Figure 2). Physical exercises were adapted due to the limitations caused by the paretic limb in the range of motion (ROM). To seated row, the paretic hand was anchored in the wrist of the nonparetic hand, while the elastic band was positioned between the palm of the paretic hand, the palm of the nonparetic hand, and the wrist. To chest press, the elastic band was anchored in the paretic side, and the abduction of the shoulder was performed according to ROM limitations. No specific changes were performed in squat on the chair exercise. The nonparetic limb executed the exercises across the full ROM.

The dynamic resistance training protocol consisted of a sequence of 3 combinations of 2 consecutive exercises (i.e., seated row and squat on the chair, vertical chest press and squat on the chair, knee extension and squat on the chair) in a dynamic manner, without intervals of absolute rest throughout the session. The concentric contractions were performed as fast as possible, while the eccentric contractions were performed within 3 s. The exercise volume was increased over the 8-week protocol, so that 3 sets of 6-8 repetitions at moderate intensity (3 to 5 points on adapted Borg Scale of 1-10 [17]) were performed in the first 4 weeks and 3 sets of 10-12 repetitions at moderate intensity were performed in subsequent weeks.

The exercise intensity was controlled according to the tension of elastic bands based on the rate of perceived exertion (RPE) method [17]. According to a study by Colado and Triplett [18], the combination of target repetitions with a subjective effort scale may be considered a valid strategy to control the intensity when RT is performed with elastic bands. The RPE was reported after the end of each set of exercise and, if the participant reported an RPE below the expectations (low intensity), the tension of the elastic band was increased (moderate intensity).

All patients were performed neurological physical therapy treatment two times per week in addition to the 8-week RT program.

### 2.4. Control Group (CG)

Patients in the CG remained performed two sessions per week over 8 weeks of a neurological physical therapy program, which consisted of physical movements that mimic basic and instrumental ADL, postural changes, and gait exercises on parallel bars.

### 2.5. Evaluations

#### 2.5.1. Functional Parameters

A researcher detailed the operational procedures, demonstrated the test, and evaluated the motor pattern of participants during each physical performance test. All participants performed a familiarization trial to ensure that they had understood the test. All tests were performed in triplicate, and the mean result was used in the final analysis. A 1 min rest was allowed between consecutive trials. Four physical tests were administered in the following order: (a) isometric handgrip of paretic and nonparetic limbs (IHGPL and IHGNPL), (b) 10 m walking test (10MWT), (c) five-repetition sit-to-stand (5XSTS), and (d) timed “up and go” (TUG).

#### 2.5.2. Isometric Handgrip of Paretic and Nonparetic Limbs

Isometric handgrip strength was measured using a Jamar® handheld hydraulic dynamometer (Sammons Preston, Bolingbrook, IL, USA). The measure was obtained with the participant seated in a chair with the shoulders abducted, elbows near the trunk and flexed at 90°, and wrists in a neutral position (thumbs up). The contralateral arm remained relaxed under the thigh. To determine handgrip strength, participants performed a maximal contraction during 3-5 s with the paretic (IHGPL) and nonparetic (IHGNPL) limbs [19]. The maximum grip strength (kgf) was taken from the digital display. The test reliability in the present study was ≥0.8 (*κ* = 0.99).

#### 2.5.3. 10 m Walking Speed (10MWT)

Walking speed was measured over 10 m. Participants were required to walk 12 m at their fastest possible pace without running. Before the evaluation, both feet of each participant remained on the starting line. The time measurement started when a foot reached the 1 m line and was stopped when a foot reached the 11 m line. The 1 m intervals at the beginning and the end of the course were used to avoid early acceleration and/or deceleration [20]. The following formula was used to calculate walking speed:

(1) $$\begin{array}{c} 10\text{MWT}=\frac{10}{\text{time to complete the test}}. \end{array}$$

The test reliability in the present study was ≥0.8 (*κ* = 0.98).

#### 2.5.4. 5-Repetition Sit-To-Stand (5XSTS)

Participants were requested to rise from a standard armless chair five times as quick as possible with arms folded across the chest. The stopwatch was started when participants raised their buttocks off the chair and was stopped when participants seated back at the end of the fifth stand [21]. The test reliability in the present study was ≥0.8 (*κ* = 0.95).

#### 2.5.5. Timed “Up and Go”

The TUG involved getting up from a chair (total height: 87 cm; seat height: 45 cm; width: 33 cm), walking three meters around a cone placed on the floor, coming back to the same position, and sitting back on the chair. The volunteer wore regular footwear, with the back against the chair, arms resting on the chair's arms, and the feet in contact with the ground. A researcher instructed the volunteer to, on the word “go,” get up, walk as fast as possible without compromising safety through the demarcation of three meters on the ground, turn, return to the chair, and sit down again. The timing was started when participants got up from the chair and was stopped when the participants back touched the backrest of the chair [22, 23]. The test reliability in the present study was ≥0.8 (*κ* = 0.95).

#### 2.5.6. Hemodynamic Parameters

*(1) Blood Pressure Measurement*. Blood pressure (BP) was measured between 08 : 00 and 10 : 00 am according to the procedures detailed in the 7th Brazilian Arterial Hypertension Guidelines [24]. Participants were instructed to refrain from exercising during the previous 48 h and from drinking caffeinated beverages and/or alcohol 24 h before the evaluation. After remained seated on a comfortable recliner chair for 15 min in a quiet room, an appropriate cuff was placed at approximately the midpoint of the participant's upper left arm. An automatic, noninvasive, calibrated, and validated arterial BP monitor (Microlife-BP 3BT0A, Microlife, Widnau, Switzerland) [24] was used to measure systolic BP (SBP), diastolic BP (DBP), and heart rate (beats per min, bpm). The double product (DP) was calculated as follows:

(2) $$\begin{array}{c} \text{DP}=\text{SBP}\times \text{heart rate}. \end{array}$$

*(2) Assessment of Heart Rate Variability (Cardiac Autonomic Modulation)*. A Polar V800 heart rate monitor (Polar Electro Oy, Kempele, Finland) was used to continuously record beat-to-beat intervals (R-R interval) with the patients in the supine position [25]. The spectrum resulting from the fast Fourier transforms modeling was derived from the highest value in one of the for 5-minute window recorded; it includes the entire signal variance, regardless of whether its frequency components appear as specific spectral peaks or as nonpeak broadband powers. The R-R interval variability was evaluated in the time and frequency domains. Spectral power for low (LF: 0.03–0.15 Hz) and high (HF: 0.15–0.4 Hz) frequency bands was calculated using power spectrum density integration within each frequency bandwidth, using a customized routine (MATLAB 6.0, Natick, MA, USA). The LF/HF ratio was calculated based on normalized LF and HF. The time domain measurements included standard deviation of the of normal sinus beats (SDNN, ms) and root mean square of successive R-R interval differences (RMSSD, ms).

The nonlinear geometric measures have been derived from the 5-minute Poincaré plot representing a diagram in which each R-R interval of tachogram is plotted against the previous R-R interval. The length of the longitudinal line is defined as the SD2 of the plot data. The length of the transverse line is defined as the SD1 of the plot data in a perpendicular direction.

#### 2.5.7. Oxidative Stress Markers

Blood samples were collected by venipuncture in heparinized vacutainers after 12 h fasting and immediately centrifuged at 4000 rpm for 5 min to separate plasma. Participants were advised to avoid foods rich in nitrates (e.g., beet, cabbage, spinach, lettuce) the day before blood collection. Protein concentration was determined according to the method described by Lowry et al. [26], using bovine albumin solution at a concentration of 1 mg/mL as the standard and 10 *μ*L samples.

Thiobarbituric acid reactive substances (TBARS), carbonyls, NADPH oxidase, hydrogen peroxide (H_2_O_2_), superoxide dismutase (SOD), and plasma nitrite analyses were conducted in accordance with Jacomini et al. [27].

### 2.6. Statistical Analysis

Data distribution and equality of variance were tested by the Shapiro-Wilk and Levene tests, respectively. Repeated measures ANOVA (followed by the Sidak post hoc test) was used to detect differences between different times of evaluations and treatments. 10MWT (s), SBP (mmHg), and HF band (ms^2^) showed irregular distribution and within- and between-group differences were analyzed using the Wilcoxon and Mann–Whitney tests, respectively. Chi-square (*χ*^2^) statistics were used to compare categorical variables. Cohen's ES *d* was calculated to assess the magnitude of the results. Delta (*∆*) values were calculated as follows:

(3) $$\begin{array}{c} ∆=\text{Mean post}-\text{Mean baseline}. \end{array}$$

The level of significance was set at alpha = 5% (*P* < 0.05), and all analyses were performed using the GraphPad Prism 7.00 (San Diego, CA).

## 3. Results

Twenty-seven volunteers were recruited and accepted to be evaluated for eligibility. Three candidates declined to participate, while two decided to engage in another exercise program, leaving a total of 22 stroke survivors who were randomized into two groups (i.e., CG [*N* = 11] or TG [*N* = 11]). There were no withdrawals from either group (Figure 1).

The baseline characteristics of the study participants are shown in Table 1. The mean time since stroke was 5 years. The mean age of the whole sample was 62.2 ± 10.8 years and the mean body mass index (BMI) value was 24.8 ± 3.0 (kg/m^2^). The most common pharmacological therapy was diuretics, followed by statins, angiotensin-converting enzyme inhibitor (ACEi), and antidiabetic agents, which can be explained by the high prevalence of hypertension and type 2 diabetes mellitus observed in our sample. No significant differences were observed among the groups.

### 3.1. Physical Function

Physical function is shown in Table 2. No significant differences were observed among the groups at baseline. After 8 weeks, TG improved 10MWT (*P* = 0.0001, Δ = −38.3%, *d* = −0.8), sit-to-stand (*P* = 0.0001, Δ = −30.6%, *d* = −1.9), and TUG tests (*P* = 0.0001, Δ = −23.0%, *d* = −0.7). In contrast, a significant reduction in IHGPL (*P* = 0.017, Δ = −24.0%, *d* = −0.3) and IHGNPL (*P* = 0.016, Δ = −16.8%, *d* = −0.4) was observed in the CG. Between-group comparisons indicated better TUG (*P* = 0.042, Δ = −28.2%; *d* = −1.2) and sit-to-stand (*P* = 0.011, Δ = −29.1%, *d* = −1.5) performances in TG when compared to CG. A larger ES classification was attributed to changes on 10MWT in TG in comparison to CG (*d* = −0.9).

### 3.2. Hemodynamic Parameters

Hemodynamic parameters are shown in Table 3. No significant differences were observed among the groups at baseline. SBP and DBP remained unchanged in both TG and CG over the experimental period. In contrast, heart rate (*P* = 0.047, Δ = −11.5, *d* = −0.6) and DP (*P* = 0.011, Δ = −13.1%, *d* = −1.4) were significantly reduced in TG in comparison with CG after 8 weeks (*P* = 0.047, Δ = −11.5, *d* = −0.6).

### 3.3. Cardiac Autonomic Modulation

**Figure 3:**
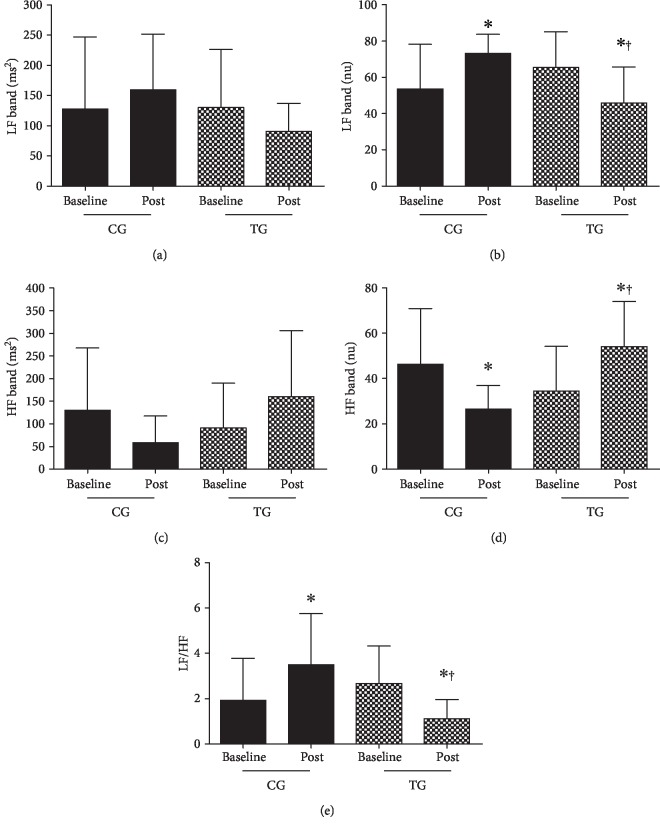
Cardiac autonomic modulation. (a) Low-frequency band (LF, ms^2^). (b) Low-frequency band (LF, nu). (c) High-frequency band (HF, ms^2^). (d) High-frequency band (HF, nu). (e) Autonomic balance (LF/HF). Data are shown as mean ± SD or median. CG: control group; TG: training group; LF: low-frequency band; HF: high-frequency band; ^∗^*P* < 0.05 in comparison to baseline; ^†^*P* < 0.05 in comparison to CG at the same moment.

Cardiac autonomic modulation parameters are shown in Table 3 and Figure 3. No significant differences were observed among the groups at baseline. SDNN (*P* = 0.0001, Δ = 39.3%, *d* = 1.0), rMSSD (*P* = 0.014, Δ = 30%, *d* = 0.70), SD1 (*P* = 0.014, Δ = 30.4%, *d* = 0.71), SD2 (*P* = 0.002, Δ = 27.3%, *d* = 0.87), LF band in ms^2^ (Figure 3(a), no difference was observed), LF band in nu (Figure 3(b)) (*P* = 0.004, Δ = −29.9%, *d* = −1.5), HF band in ms^2^ (Figure 3(c), no difference was observed), HF in nu (Figure 3(d)) (*P* = 0.003, Δ = 57.0%, *d* = 1.5), and LF/HF ratio (Figure 3(e)) (*P* = 0.004, Δ = −1.56%, *d* = −1.5) were improved in response to exercise when compared to baseline values and CG. On the other hand, elevated LF (nu) (*P* = 0.006, Δ = 36.7%, *d* = 1.5) and reduced HF (nu) (*P* = 0.006, Δ = −42.6%, *d* = −1.5) were observed in CG in comparison to baseline.

### 3.4. Oxidative Stress Markers

**Figure 4:**
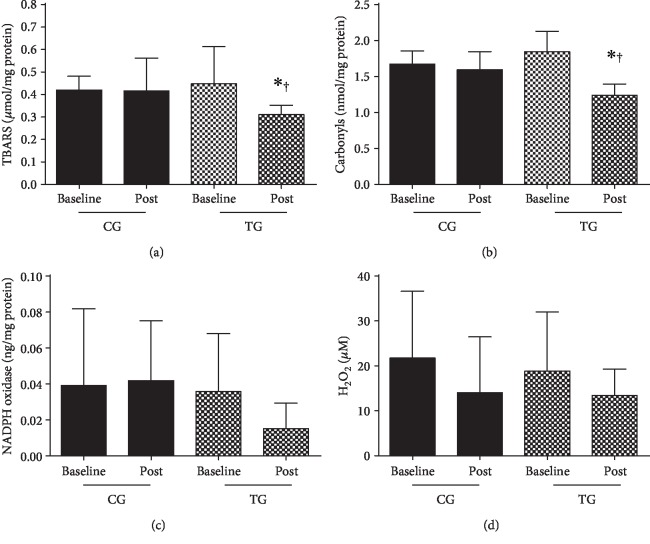
Oxidant markers. (a) Thiobarbituric acid reactive substances. (b) Carbonyls. (c) NADPH oxidase. (d) Nitrite peroxide (H_2_O_2_). Data are shown as mean ± SD. CG: control group; TG: training group. ^∗^*P* < 0.05 in comparison to baseline; ^†^*P* < 0.05 in comparison to CG at the same moment.

**Figure 5:**
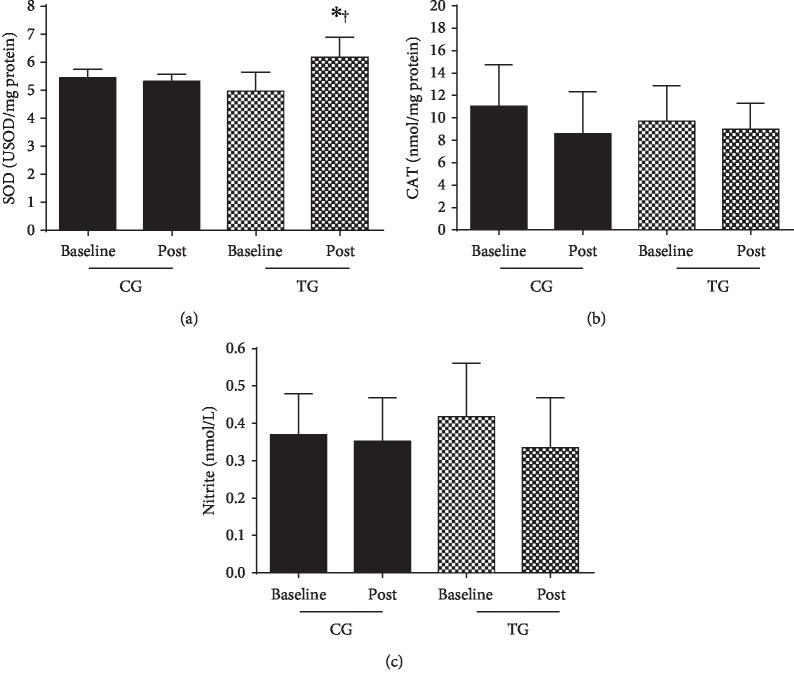
Antioxidant markers and plasma nitrite concentration. (a) Superoxide dismutase (SOD). (b) Catalase (CAT). (c) Nitrite. Data are shown as mean ± SD. CG: control group; TG: training group. ^∗^*P* < 0.05 in comparison to baseline; ^†^*P* < 0.05 in comparison to CG at the same moment.

Oxidative stress markers are shown in Figures 4 and 5. No significant differences were observed among the groups at baseline. TG improved TBARS (Figure 4(a); *P* = 0.0428), carbonyls (Figure 4(b); *P* < 0.0001), and SOD (Figure 5(a); *P* = 0.0001) levels in comparison to baseline and CG. CAT (Figure 5(b); *P* = 0.3219) and nitrite (Figure 5(c); *P* = 0.5662) levels were unchanged over the experimental period.

## 4. Discussion

The main findings of the present study indicate that 8-week dynamic resistance training protocol with elastic bands improved physical function, hemodynamic parameters, autonomic modulation, and oxidative markers in stroke patients. In contrast, a significant reduction in upper-limb muscle strength (i.e., IHGPL and IHGNPL) was observed in CG.

Although many studies [28–30] have investigated the effects of RT on the physical of stroke survivors, results are still not conclusive. Supporting our findings, Hill et al. [30] observed significative improvements in the gait ability and TUG performance of stroke patients after a lower-limb high-intensity RT protocol. On the other hand, no RT effects in gait velocity were reported in other protocols [28, 29, 31].

A possible explanation for the differences among the studies may that concentric contractions in the present study were performed as fast as possible, given that many aspects of the physical function seem to be more closely associated with muscle power than muscle strength [32] and greater gains in physical performance have been observed after power training in comparison to traditional RT [32–34].

These findings have important clinical and public health implications since better physical performance in patients with stroke is associated with a higher likelihood of social integration, independence to perform ADL, and better quality of life [35–37]. Besides that, stroke survivors with poor physical function are more likely to experience a recurrent stroke and die in a short-term interval after the first event in comparison with those with proper physical function [38, 39].

Another significant finding of the present study is regarding the importance of upper-limb resistance exercises to stroke survivors since IHGPL and IHGNPL were significantly reduced in CG, while it remained unchanged in TG. IHG has been used as an essential measurement of muscle strength, and it is well-accepted as part of the assessment of sarcopenia [40, 41]. Nevertheless, IHG is strongly associated with upper-limb muscle strength in stroke, and it has a critical role in the physical performance of this population [3, 38].

No significant differences in SBP (Δ = −10.41 mmHg) and DBP (Δ = −8.16 mmHg) were observed between TG and CG after 8 weeks. These findings are supported by prior studies that observed reduced blood pressures in hypertensive people after exercise training [42–44] and indicate that our exercise protocol may be associated with a significant reduction in cardiovascular and restroke risk [42, 45].

A recent meta-analysis of 12 studies evaluated the effects of aerobic exercises on blood pressure values of stroke survivors and found reductions of 4.3 mmHg and 2.5 mmHg in SBP and DBP, respectively, after the intervention [43]. Similarly, most randomized clinical trials investigating pharmacological therapy showed blood reductions of approximately 5 mmHg for SBP and 4 mmHg for DBP [39, 45]. Therefore, RT effects on blood pressure of stroke survivors are favorably similar or even more substantial than the impact of aerobic exercise and pharmacological therapy, suggesting that RT may be an essential tool in the management of blood pressure and cardiovascular risk in patients with stroke [42–44].

Cardiac autonomic modulation and oxidative stress markers were investigated as two possible mechanisms associated with blood pressure lowering in response to RT. Our findings suggest that RT improved vagal modulation (i.e., rMSSD, SD1, SD2, HF) and sympathovagal balance (i.e., LF/HF ratio).

Notably, changes in cardiac autonomic modulation are not commonly observed in response to RT protocol [44, 46, 47], while substantial evidence have reported this phenomenon after aerobic training [47, 48]. A hypothesis that may account for our findings lies in the dynamic characteristic of our RT protocol, in which absolute rest intervals between sets and exercises were not provided, so that the cardiovascular and neuromuscular systems were stimulated simultaneously throughout each session [49]. In this context, our RT protocol had an aerobic component able to improve cardiac autonomic modulation, as usually occurs with the practice of aerobic training [47, 48].

Although exercise may acutely increase reactive oxygen species, a compensatory mechanism seems to occur after chronic exercise training, in which exercise training upregulatesthe amount and efficiency of antioxidant enzymes [48]. Findings of the present study support this hypothesis by observing increased SOD levels and reduced TBARS and carbonyls levels after RT.

There are some limitations in the present study which should be addressed by future investigations to confirm and expand our findings, as the short period of intervention and the absence of muscle strength assessments of all trained muscle groups. Our sample size is also a limitation of our study since a post hoc sample size calculation estimated that about 14 participants in each group would be needed to detect improvements in physical function, hemodynamic parameters, and oxidative markers considering within- and between-group comparisons, with 80% power at the 5% significance level. Finally, unexpected changes on upper-limb muscle strength (i.e., IHGPL and IHGNPL) and autonomic modulation (i.e., RMSSD and SD1) were observed in CG. Although pain (Terwee et al., 2006), white matter lesions (Zerna et al., 2018), or even the presence of other comorbidities may explain the substantial declines in IHGPL and IHGNPL, as well as psychosocial stress may impact cardiac modulation (Lucini et al., 2005), future studies are still needed to confirm our findings.

## 5. Conclusions

Our findings indicate that an 8-week dynamic resistance training protocol with elastic bands improved physical function, hemodynamic parameters, autonomic modulation, and oxidative stress markers in chronic ischemic stroke survivors.

## Figures and Tables

**Table 1:** Baseline clinical characteristics of participants.

	CG (*n* = 11)	TG (*n* = 11)	*P* value
Age (years)	60.5 ± 13.2	66.4 ± 10.1	0.334
Body mass index (kg/cm^2^)	26.0 ± 3.2	25.4 ± 2.9	0.089
Women (%)	63.6	54.5	0.120
Poststroke duration (years)	4.9 ± 4.2	6.6 ± 5.0	0.120
Paretic side (left) (%)	90.9	54.5	0.987
Basic functional independence (Barthel Index)	90.0 ± 6.3	87.3 ± 11.9	0.350
Baecke Habitual Physical Activity Questionnaire	3.8 ± 0.6	3.2 ± 0.4	0.884
Associated comorbidities (%)			
Hypertension	90.9	90.9	1.000
T2DM	54.5	56.4	0.916
Medications (%)			
ACE inhibitors	70.3	74.5	0.842
HMG-CoA reductase inhibitor	88.2	85.3	0.859
Diuretics	77.8	75.9	0.898
Acetylsalicylic acid	45.3	47.8	0.973
Antidiabetics	55.7	58.2	0.948

Data are shown as mean ± SD. CG: control group; TG: training group; T2DM: diabetes mellitus type II; ACE: angiotensin-converting enzyme; HMG-CoA: 3-hydroxy-3-methylglutaryl coenzyme A reductase.

**Table 2:** Physical function at baseline and after 10 weeks.

Variables	CG (*n* = 11)		*∆* (ES)	TG (*n* = 11)		*∆* (ES)
	Baseline	Post		Baseline	Post	
IHGPL (kgf)	10.4 ± 8.9	7.9 ± 7.7∗	-2.5 (0.3)	13.8 ± 10.7	13.9 ± 10.0	0.1 (-0.0)
IHGNPL (kgf)	28.5 ± 13.9	23.7 ± 10.8∗	-4.8 (0.4)	28.5 ± 7.3	28.1 ± 8.0	-0.4 (0.1)
10MWT (s)	14.5 (10.4–31.5)	13.5 (10.0–32.0)	0.7 (-0.1)	13.8 (10.0–42.4)	10.2 (7.9–22.2)^∗^^†^	-6.4 (0.8)
Sit-to-stand (s)	15.1 ± 2.9	14.4 ± 2.4	-0.7 (0.3)	15.7 ± 3.0	11.3 ± 1.7^∗^†	-4.4 (1.9)
TUG (s)	22.2 ± 9.3	22.0 ± 7.1	-0.2 (0.0)	19.2 ± 8.3	14.1 ± 5.6^∗^†	-5.1 (0.7)

SD: standard deviation of the mean; ES: effect size; CG: control group; TG: training group; IHGPL: isometric handgrip of the paretic limb; IHGNPL: isometric handgrip of the nonparetic limb; 10MWT: 10-meter walking speed; TUG: timed up and go. Data are shown as mean ± SD or median; ^∗^*P* < 0.05 vs. baseline; ^†^*P* < 0.05 vs. CG.

**Table 3:** Hemodynamic and autonomic parameters at baseline and after 10 weeks.

Variables	CG (*n* = 11)		*∆* (ES)	TG (*n* = 11)		*∆* (ES)
	Baseline	Post		Baseline	Post	
Hemodynamics						
SBP (mmHg)	133 (94-139)	129 (99-140)	0.3 (-0.0)	130 (94–139)	121 (95–137)	-5.6 (0.4)
DBP (mmHg)	79.2 ± 11.9	79.8 ± 10.9	0.6 (-0.1)	72.5 ± 14.4	71.6 ± 12.4	-0.9 (0.1)
HR (bpm)	74.8 ± 14.4	76.5 ± 11.2	1.7 (-0.1)	71.5 ± 11.9	65.1 ± 9.5^†^	-6.4 (0.6)
DP (mmHg × bpm)	9938.1 ± 2226.8	9949.5 ± 1852.6	10.9 (0)	8890.6 ± 1607.1	7722.0 ± 1375.2^†^	-1168.0 (0.8)
Autonomics						
Time domain indexes						
SDNN (ms)	20.4 ± 8.2	19.4 ± 6.8	-1.0 (0.1)	23.9 ± 7.5	33.3 ± 10.8^∗^^†^	9.4 (-1.0)
rMSSD (ms)	17.2 ± 9.9	12.7 ± 5.2	-4.5 (0.6)	16.6 ± 8.3	23.7 ± 11.6^∗^^†^	7.1 (-0.7)
Nonlinear indexes						
SD1 (ms)	12.2 ± 7.0	9.0 ± 3.7	-3.2 (0.6)	11.7 ± 5.8	16.8 ± 8.2^∗^^†^	5.1 (-0.7)
SD2 (ms)	25.7 ± 10.5	25.7 ± 9.6	0 (0)	31.3 ± 10.5	43.1 ± 15.9^∗^^†^	11.8 (-0.9)

SD: standard deviation of the mean; ES: effect size; CG: control group; TG: training group; SBP: systolic blood pressure; DBP: diastolic blood pressure; DP: double product; SDNN: selected standard deviation of normal R-R intervals; rMSSD: square root of the mean squared differences between adjacent normal R-R intervals, expressed in ms; SD1: short variation of R-R interval; SD2: represents HRV in long-term records. Data are shown as mean ± SD or median; ^∗^*P* < 0.05 vs. baseline; ^†^*P* < 0.05 vs. CG.

## Data Availability

The data used in this study is available from the corresponding author upon request.
